# Correction to New diagnostic criteria (2023) for slowly progressive type 1 diabetes (SPIDDM): Report from Committee on Type 1 Diabetes of the Japan Diabetes Society (English version)

**DOI:** 10.1111/jdi.14190

**Published:** 2024-03-27

Shimada A, Kawasaki E, Abiru N, Awata T, Oikawa Y, Osawa H, Kajio H, Kozawa J, Takahashi K, Chujo D, Noso S, Fukui T, Miura J, Yasuda K, Yasuda H, Imagawa A, Ikegami H. New diagnostic criteria (2023) for slowly progressive type 1 diabetes (SPIDDM): Report from Committee on Type 1 Diabetes of the Japan Diabetes Society (English version). J Diabetes Investig. 2024 Feb;15:254–257.

In the original publication of the article, under the discussion section, in the following paragraph “Moreover, regarding the concept of …”, “for” should be replaced with “at” in the third point.

The correct sentence should read as follows “(3) insulin treatment required at more than 6 months after the diagnosis of diabetes.”

Similarly, under the same section, in the following paragraph “According to the IDS diagnostic criteria for…”, “for” should be replaced with “at” in a few occurrences.

The correct sentences should read as follows “According to the IDS diagnostic criteria for LADA, insulin treatment is required at more than 6 months [4] after the diagnosis of diabetes. However, considering the diagnostic criteria of AT1D [6], we described the necessity of insulin treatment at more than 3 months in the new criteria of SPIDDM (2023) and added a comment on the necessity of insulin treatment at more than 6 months after diagnosis of diabetes in typical cases.”

Finally, in Table 1, on third factor, “for” should be replaced with “at”. The correct sentence should read as follows “(3) Gradual reduction in insulin secretion overtime, requirement of insulin treatment at more than 3 months^b^ after diagnosis of diabetes, and severe endogenous insulin deficiency (fasting serum C‐peptide immunoreactivity <0.6 ng/mL) at the last observed time point”.

We apologize for this error.

